# Gastric Metastasis from Small Cell Lung Cancer: An Unusual Site of Metastatic Spread: A Case Report

**DOI:** 10.2174/0115734056478330260701111121

**Published:** 2026-07-14

**Authors:** Junhan Qu, Qifan He, Xingxing Zhu, Bingfeng Qiu

**Affiliations:** 1 Department of Gastroenterology, Zhoushan Hospital, Zhoushan, Zhejiang, China; 2 Department of Radiology, Affiliated Haining Hospital of Jiaxing University, Haining People's Hospital, Jiaxing, Zhejiang, China; 3 Department of Pulmonary and Critical Care Medicine, Affiliated Haining Hospital of Jiaxing University, Haining People's Hospital, Jiaxing, Zhejiang, China

**Keywords:** Small cell lung cancer, Gastric metastasis, Computed tomography, Gastroscopy, Endoscopic ultrasound, Lung neuroendocrine tumor

## Abstract

**Background:**

Small Cell Lung Cancer (SCLC) is a lung neuroendocrine tumor with high invasiveness and early extensive metastasis. The most common metastatic sites of SCLC include the brain, bone, liver, and adrenal gland, but gastric metastasis is uncommon. We report a case of SCLC metastasizing to the stomach and discuss its imaging findings, in order to enhance clinical understanding and emphasize the key role of imaging in identifying such uncommon metastasis.

**Case Presentation:**

The patient was a 70-year-old male with no smoking history and was hospitalized due to dizziness and fatigue. Cranial and chest Computed Tomography (CT) showed nodules in the upper lobe of the left lung and intracranial space-occupying lesions, and the diagnosis of SCLC was confirmed by lung biopsy. Positron emission tomography-computed tomography further confirmed the presence of multiple metastases. A few months later, the reexamination found that the lung lesions progressed and the symptoms of abdominal distension occurred. Abdominal CT revealed a soft tissue mass in the fundus of the stomach, and endoscopic ultrasound further confirmed that it was a hypoechoic mass of submucosal origin. After gastroscopy combined with laparoscopic resection, the postoperative pathological and immunohistochemical findings supported the diagnosis of gastric metastasis from SCLC.

**Conclusion:**

This case highlights that although gastric metastasis from SCLC is unusual, we should be alert to the possibility of gastric imaging abnormalities during follow-up. Comprehensive imaging, endoscopy, and pathological examination are essential for the diagnosis.

## 1 INTRODUCTION

Small cell lung cancer (SCLC) is a lung cancer with high invasiveness and high lethality, and it accounts for about 15% of lung cancer. Its five-year survival rate is about 7% [1, 2]. SCLC grows rapidly and is prone to distant metastasis. The most common metastatic sites are the brain, bone, liver, and adrenal glands [3]. In addition, SCLC can also be transferred to some unusual sites, such as the thyroid [4], heart [5], pancreas [6], extraocular muscles [7], and tonsils [8], along with the stomach, which is also an unusual metastatic site of SCLC [9].

In the 1980s, a study conducted autopsy analysis on 423 patients with primary lung cancer and found that 58 patients (14%) had gastrointestinal metastasis and only 8 cases (13.8%) were gastric metastasis [10]. Among these 58 patients, SCLC was the least common tissue type, accounting for 11 cases (19.0%) [10]. At the beginning of the 21^st^ century, Kim *et al*. reported a lower incidence of lung cancer in the gastrointestinal tract, about 0.19% (10/5239). Among these, only one case involved gastric metastasis, and it occurred in the only patient with SCLC [11]. According to our understanding, there are only a few clinical reports on gastric metastasis from SCLC. Up to now, more than 20 cases of gastric metastasis of SCLC have been reported.

Therefore, we report a case of SCLC with gastric metastasis confirmed by postoperative pathology and immunohistochemistry. This report aims to explore the value and characteristics of imaging methods for diagnosing gastric metastasis from SCLC, using detailed imaging findings.

## 2 CASE PRESENTATION

The patient, a 70-year-old male, visited the emergency department of our hospital on July 29, 2023, due to dizziness and fatigue for 4 days. Cranial Computed Tomography (CT) showed a right frontoparietal lobe mass with surrounding brain tissue edema and low-density foci in the right cerebellar vermis (Fig. **1A** and **B**). At the same time, the chest CT showed two nodular high-density shadows in the upper lobe of the left lung (Fig. **1C** and **D**). He was admitted for further evaluation. No obvious abnormalities were found on physical examination at admission.

CT-guided percutaneous lung biopsy of left upper lobe nodules was performed on August 2, 2023. Histopathology confirmed Small Cell Lung Cancer (SCLC). Immunohistochemistry results were positive for synaptophysin (Syn), CD56, thyroid transcription factor-1 (TTF-1), cytokeratin (CK), and Ki-67 (90%), but negative for chromogranin A (CgA), P40, P63, and Napsin A (Fig. **2A**-**C**). Malignant nodules located in the apicoposterior segment of the left upper lobe were identified by a follow-up Positron Emission Tomography-Computed Tomography (PET-CT) scan. It also identified metastases in the T2 spinous process, left hilar lymph nodes, and multiple intracranial sites. In addition, PET-CT demonstrated a small hypermetabolic nodule posterior to the gastric fundus and cardia measuring approximately 12.0mm, with an SUVmax of 3.6 on conventional imaging and 9.2 on delayed imaging (Fig. **3A**-**C**). At this stage, this nodule was considered more likely to represent a metastatic lymph node adjacent to the gastric fundus and cardia, rather than definite gastric metastasis. Based on these findings, a diagnosis of extensive-stage SCLC was established.

On August 18, 2023, the patient began first-line combination therapy with atezolizumab, etoposide, and cisplatin, administered every three weeks. On September 11, 2023, cranial irradiation (40 Gy in 20 fractions) was performed. Due to severe fatigue after the first cycle, chemotherapy was stopped. Starting from the second cycle, atezolizumab monotherapy was used for maintenance therapy. As of February 9, 2024, 8 cycles had been completed. Follow-up imaging showed that the left lung lesions and intracranial lesions initially decreased but remained stable thereafter, with an overall therapeutic effect of stable disease.

On April 5, 2024, the patient presented with abdominal distension. An abdominal CT showed a round-like soft-tissue density shadow (about 28 × 35 mm) in the upper part of the gastric fundus. The stomach was compressed, and slight thickening of the adjacent gastric wall was noted (Fig. **4A**). Gastroscopy performed on April 11 revealed a submucosal protuberance at the cardia. Subsequent endoscopic ultrasound (EUS) identified a hypoechoic mass measuring approximately 35.5 × 24.5 mm, with punctate hyperechogenicity inside, appearing to originate from the fourth layer, which supported a submucosal lesion (Fig. **4B** and **D**). A contrast-enhanced abdominal CT on April 13 confirmed a well-defined, rounded, low-density lesion (approximately 32 mm in diameter) in the hepatogastric space, exhibiting mild enhancement (Fig. **4C**). Excluding contraindications, gastroscopy combined with laparoscopic gastrectomy was performed on April 16, 2024. On April 26, 2024, the postoperative pathology was consistent with metastatic neuroendocrine carcinoma (small cell) with necrosis. Immunohistochemistry results were positive for CgA, Syn, CD56, CK, and Ki-67 (90%), but negative for P40, P63, and CK20 (Fig. **2D**-**F**).

Unfortunately, the patient had sudden chest tightness and shortness of breath on May 30, 2024, during his recuperation at home and was subsequently admitted to the hospital. Acute coronary syndrome and heart failure were considered, and the patient was transferred to a specialist for further treatment. After 2 months of treatment, the patient’s family gave up further treatment and discharged him from the hospital due to deterioration of his condition, and he died shortly after discharge.

## 3 DISCUSSION

SCLC is a highly malignant tumor of neuroendocrine origin with rapid growth and early widespread metastasis. Common metastatic sites include the brain, bone, liver, and adrenal gland; however, gastrointestinal metastasis is rare. Studies have shown that the incidence of gastrointestinal metastasis of lung cancer found at autopsy is about 4.7%-14%, whereas the clinical incidence is about 1.77%, with gastric metastasis being even less common [10, 14, 15].

The clinical manifestations of gastric metastasis from SCLC are extremely atypical. Some patients have no obvious symptoms, while some patients can show epigastric pain, abdominal distension, nausea, vomiting, weight loss, anemia, and melena [13, 16]. In severe cases, SCLC gastric metastasis could also cause complications such as gastric perforation or bleeding [17, 18]. PET-CT not only plays an important role in staging and radiotherapy planning for small cell lung cancer, but also in detecting occult metastatic lesions, especially in the early diagnosis of metastatic disease [19, 20]. Unfortunately, PET-CT at initial diagnosis revealed small hypermetabolic nodules posterior to the gastric fundus and cardia. These were initially interpreted as metastatic lymph nodes, leading to an overlooked diagnosis of gastric metastasis.

In previously reported cases, the metastatic route of gastric involvement in SCLC had not been clearly demonstrated. Namikawa *et al*. and Chen *et al*. showed that gastric metastasis can occur *via* hematogenous, lymphatic, direct invasion, or peritoneal dissemination, among which hematogenous and lymphatic metastasis are the most common [21, 22]. Peng *et al* and Gao *et al*. also discussed hematogenous dissemination as an important possible mechanism, but the exact route remains uncertain [12, 23]. In contrast, in our patient, a hypermetabolic nodule near the gastric fundus and cardia was detected on early PET-CT before the gastric wall-related lesion became clinically apparent. Because this lesion was initially considered more likely to represent a metastatic lymph node near the gastric fundus and cardia, and gastric involvement was later confirmed by CT, EUS, and pathological examination, lymphatic spread was considered more likely in this case. However, hematogenous dissemination cannot be completely excluded.

Pathological and immunohistochemical examination is the key to the diagnosis. Typical SCLC cells are diffusely arranged, small in size, with scant cytoplasm, fine nuclear chromatin, and indistinct nucleolus, often accompanied by necrosis and high nuclear division [24]. Syn and CD56 are common neuroendocrine markers in immunohistochemistry, while TTF-1 positivity can suggest pulmonary origin [12, 13, 18]. In this case, the cells under Hematoxylin and Eosin (H&E) staining of primary lung and gastric metastases were consistent with the typical morphology of SCLC. Both tumor sites were positive for Syn and CD56 and exhibited a high Ki-67 proliferation index. Taken together, these findings are consistent with a pulmonary primary tumor and indicate that the gastric lesion represents metastatic disease.

Gastric metastasis from SCLC is usually identified in advanced stages of the disease and is associated with limited survival. Previous studies have reported a mean survival of less than 100 days in patients with SCLC involving the gastrointestinal tract [12, 25]. Gao *et al*. described a 66-year-old male patient with gastric metastasis secondary to SCLC who received first-line chemotherapy with etoposide and cisplatin; despite treatment, the patient died approximately three months after diagnosis [23]. In another report, Casella *et al*. documented a male patient who already had multiple organ metastases at the time gastric involvement was detected. Only supportive care was provided, and the patient died within one month [26]. Therefore, gastric metastasis is more appropriately regarded as a manifestation of advanced aggressive disease and poor prognosis.

In lung cancer, emerging technologies such as 3D lung models and 3D printing may assist in surgical planning [27]. However, for SCLC, systemic therapy is still widely considered the main treatment method. In specific situations, surgical intervention can still be considered, such as the need for pathological confirmation, symptom relief, or management of tumor-related complications such as gastrointestinal bleeding [9, 28]. In this case, the surgery was mainly performed to determine a clear diagnosis and relieve abdominal distension. The patient not only received an accurate pathological diagnosis, but also experienced relief of abdominal distension after gastric tumor surgery. In addition, surgical resection may help reduce the potential risk of subsequent local complications, such as gastrointestinal bleeding or perforation. Unfortunately, the patient developed acute coronary syndrome 1.5 months after surgery, resulting in a sharp deterioration of physical status and lost the opportunity to continue systemic treatment.

## CONCLUSION

During follow-up of SCLC patients, if new gastric imaging abnormalities or gastrointestinal symptoms develop, the possibility of metastasis should be considered, and endoscopic examination and biopsy should be performed promptly. Multimodal integration of imaging, pathology, and immunohistochemistry is of decisive significance for definite diagnosis.

## Figures and Tables

**Fig. (1):**
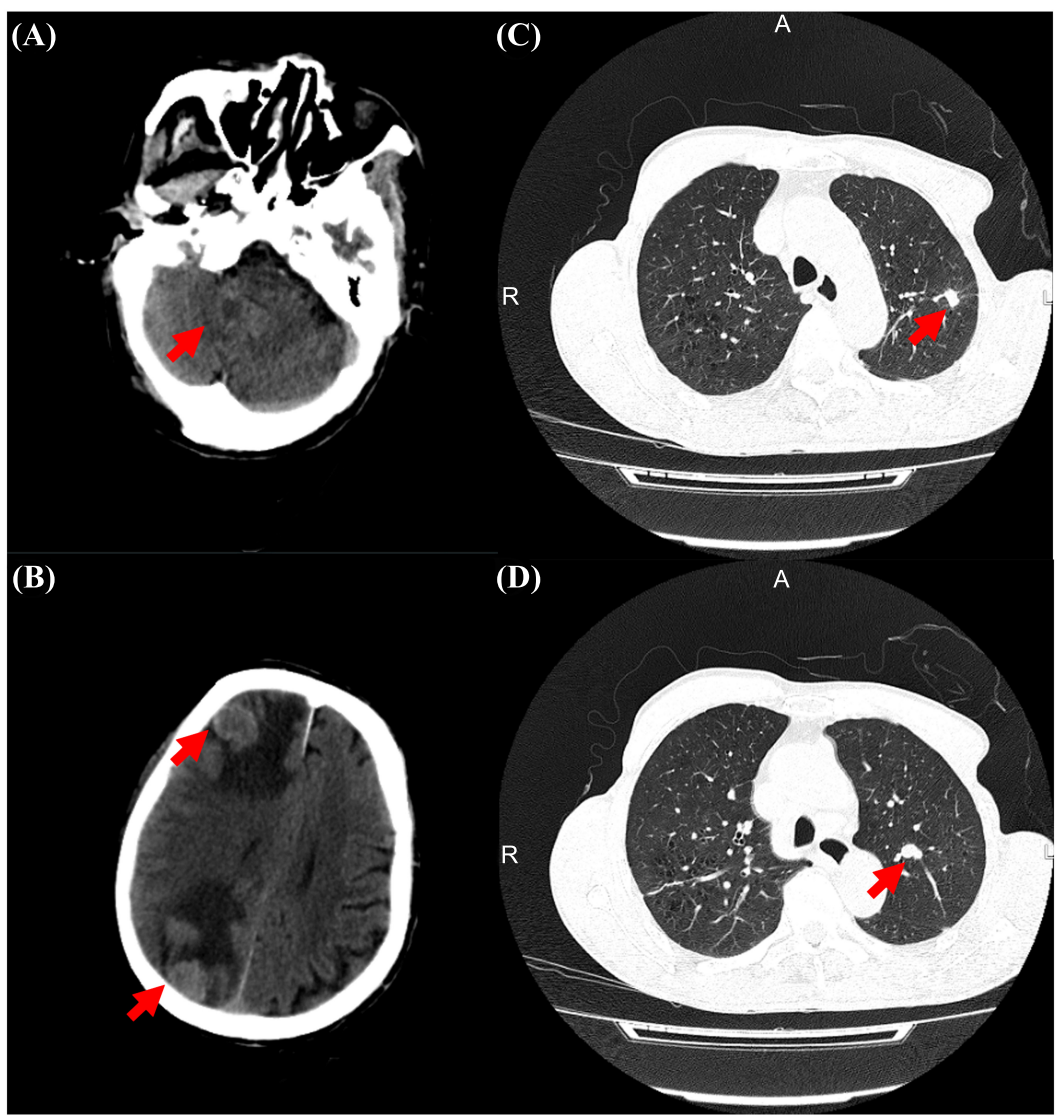
Initial cranial and chest CT findings. (Red arrows indicate malignant lesions). (**A**-**B**) On July 29, 2023, cranial CT showed a nodular high-density shadow in the right frontoparietal lobe with a clear boundary, measuring about 30 × 20 mm, accompanied by a large surrounding low-density edema area. In addition, a nodular low-density lesion was seen in the right cerebellar tonsillar region, with an unclear boundary, measuring about 13 × 10 mm. (**C**-**D**) On July 29, 2023, chest CT showed two nodular high-density shadows in the left upper lobe, the larger one was about 10 × 7mm, the boundary is clear, lobulation can be seen, and strip shadow can be seen around.

**Fig. (2):**
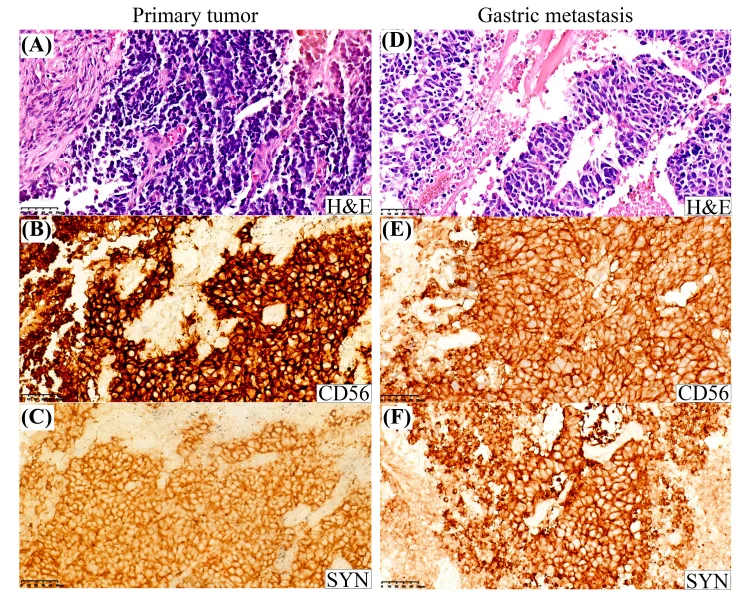
Histopathological and immunohistochemical characterization of the primary lung tumor and gastric lesion. (**A**) Hematoxylin and eosin (H&E) staining (40×) of the left upper lung biopsy specimen obtained on August 9, 2023, reveals tumor cells arranged in diffuse sheets. The cells exhibit characteristic features of small cell carcinoma, including scanty cytoplasm and hyperchromatic nuclei with finely granular chromatin. (**B**, **C**) Immunohistochemical staining of the lung biopsy specimen demonstrates positive expression for CD56 (B; 40×) and Syn (C; 40×). (**D**) H&E staining (40×) of the gastric resection specimen from April 26, 2024, displays a dense infiltration of small tumor cells with morphological features virtually identical to those of the primary lung lesion. (**E**, **F**) Immunohistochemical staining of the gastric resection specimen demonstrates positive expression for CD56 (E; 40×) and Syn (F; 40×).

**Fig. (3):**
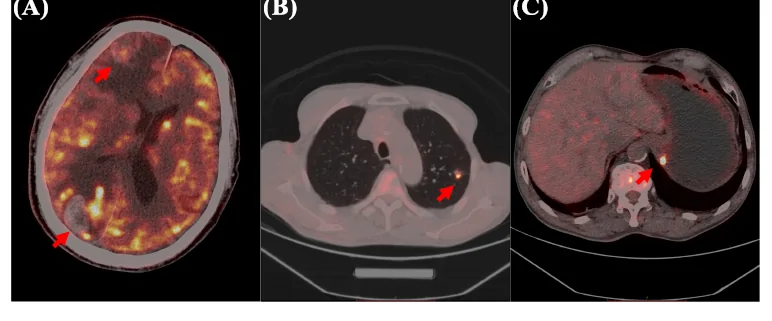
PET-CT assessment of systemic malignant lesions. (**A**) Images reveal a hyperdense nodular lesion in the right frontoparietal region; the larger component is located in the parietal lobe (maximum cross-section: approx. 19.0 × 26.7 mm) and is surrounded by an extensive area of fluid-density edema exhibiting reduced fludeoxyglucose (FDG) uptake. Additionally, the right occipital lobe shows focal patch-like hypermetabolism (SUVmax = 24.2), and the right cerebellar hemisphere presents a ring-like, slightly hyperdense nodule (approx. 15.9 × 20.6 mm). (**B**) A small nodule, about 9.9 × 7.1mm in size, was seen in the posterior segment of the upper lobe apex of the left lung. A focus of increased FDG uptake was seen, about 5.5 mm in diameter. The SUVmax of dual phase imaging was 7.8. (**C**) Gastric antrum wall showed thickening, and FDG uptake was mildly increased, SUVmax=1.6. A small nodule with increased FDG uptake was seen behind the gastric fundus and cardia, with a diameter of about 12.0mm. SUVmax was 3.6 on conventional imaging and 9.2 on delayed imaging.

**Fig. (4):**
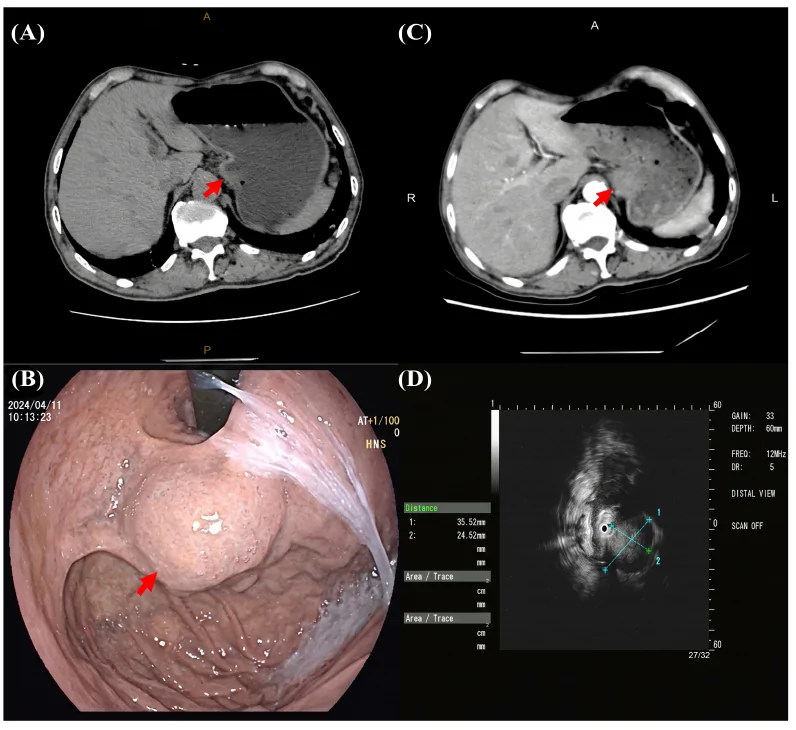
Multimodal imaging findings of gastric metastasis. (Red arrows indicate malignant lesions). (**A**) On April 5, 2024, abdominal CT showed a round soft-tissue density lesion (about 28 × 35 mm) in the upper part of the gastric fundus. The stomach was compressed, and slight thickening of the adjacent gastric wall was observed. (**B**) On April 11, 2024, routine gastroscopy revealed a submucosal bulge with a smooth surface at the cardia. (**C**) On April 13, 2024, contrast-enhanced abdominal CT showed a well-defined circular low-density lesion in the hepatogastric space, measuring about 32 mm in diameter, with mild enhancement after contrast administration. (**D**) On April 11, 2024, endoscopic ultrasound showed a hypoechoic mass with punctate hyperechogenicity inside, originating from the fourth layer, measuring approximately 35.5 × 24.5 mm.

## Data Availability

The data and supportive information are available within the article.

## LIST OF ABBREVIATIONS

SCLC: Small cell lung cancer
CT: Computed tomography
PET-CT: Positron emission tomography-computed tomography
Syn: Synaptophysin
TTF-1: Thyroid transcription factor-1
CK: Cytokeratin
CgA: Chromogranin A
MRI: Magnetic resonance imaging
FDG: Fluorodeoxyglucose
EUS: Endoscopic ultrasound
H&E: Hematoxylin and eosin

## STANDARDS OF REPORTING

CARE guidelines were followed.
